# False Negative ^99m^Tc-DPD Scintigraphy in pVal50Met (Val30Met) Hereditary Transthyretin Amyloidosis

**DOI:** 10.1016/j.cjco.2023.11.017

**Published:** 2023-11-21

**Authors:** Betim Redzepi, Christel H. Kamani, Niccolo Maurizi, Marie Théaudin, John Prior, Pierre Monney

**Affiliations:** a Department of Cardiology, Lausanne University Hospital (CHUV), Lausanne, Switzerland; b Department of Nuclear Medicine and Molecular Imaging, Lausanne University Hospital (CHUV), Lausanne, Switzerland; c Department of Neurology, Lausanne University Hospital (CHUV), Lausanne, Switzerland; d University of Lausanne (UNIL), Faculty of Biology and Medicine, Lausanne, Switzerland

**Hereditary ATTR amyloidosis (hATTR) is a rare genetic disorder with autosomal dominant transmission, resulting from a point mutation in the transthyretin (TTR) gene. Symptoms are related to the deposition of TTR fibrils (amyloid deposits) in various tissues, including peripheral nerves and the heart. Cardiac symptoms include myocardial hypertrophy and conduction disorders. Among the more than 100 recognized TTR mutations, the pVal50Met (Val30Met) variant is the most prevalent worldwide with early onset of the disease. Assessment of cardiac amyloid infiltration primarily relies on cardiac imaging including echocardiography, cardiac magnetic resonance and** ^**99m**^ **Tc-PYP/DPD/HMDP scintigraphy. Scintigraphy is reported as particularly sensitive, allowing early diagnosis.** ^1^

## Case

We report the case of a Portuguese man, carrier of the pVal50Met (Val30Met) TTR mutation. At his first visit at our neurology clinic at age 38, he was asymptomatic, with normal clinical examination results, normal nerve conduction (NCS), and no autonomic dysfunction. The patient did not attend the planned cardiology visit. He came back 2 years later, complaining of neuropathic pain and mild sensory deficits in both feet and impotency. Clinical examination and NCS showed a sensory-motor axonal length-dependent neuropathy, bilateral carpal tunnel syndrome, and mild orthostatic hypotension. Again, the patient did not show up at the planned cardiology visit.

**Figure 1:**
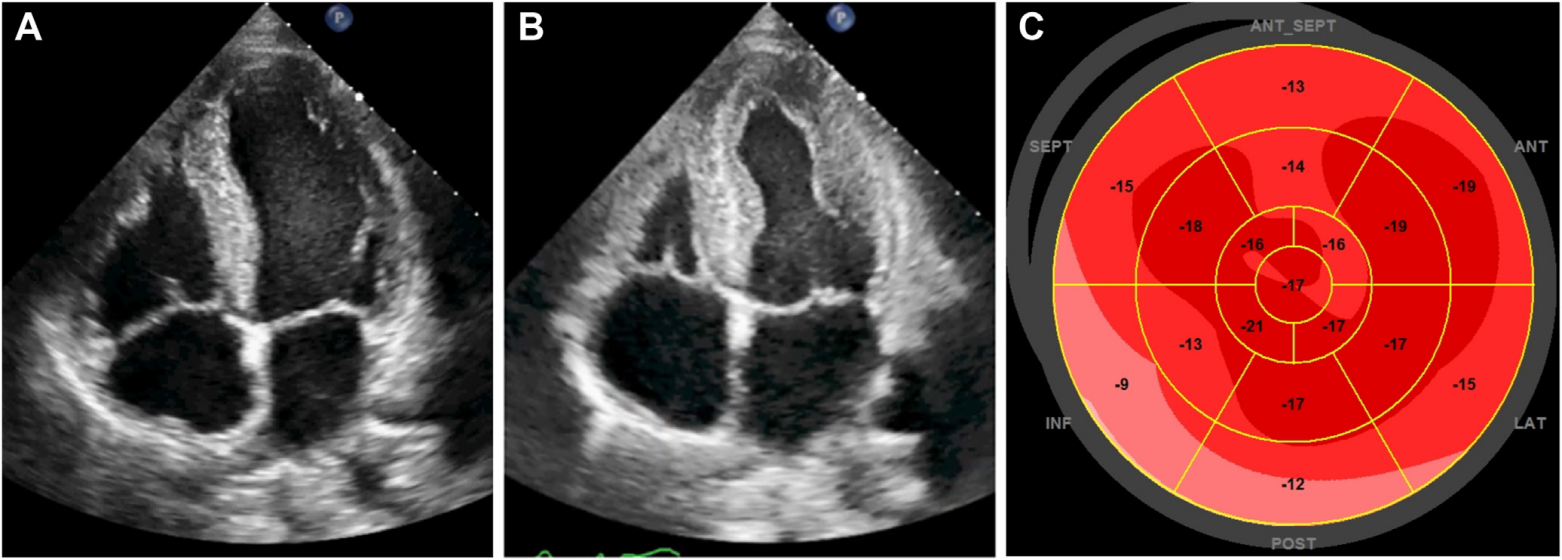
Echocardiographic apical 4-chamber view in diastole (**A**) and systole (**B**). Maximal wall thickness was 12 mm, and left ventricular ejection fraction was 53%. Speckle-tracking imaging showed a moderate reduction in left ventricular longitudinal function, with a global longitudinal strain of –14.6%. The reduction in longitudinal shortening was most prominent at the left ventricular base (**C**).

**Figure 2:**
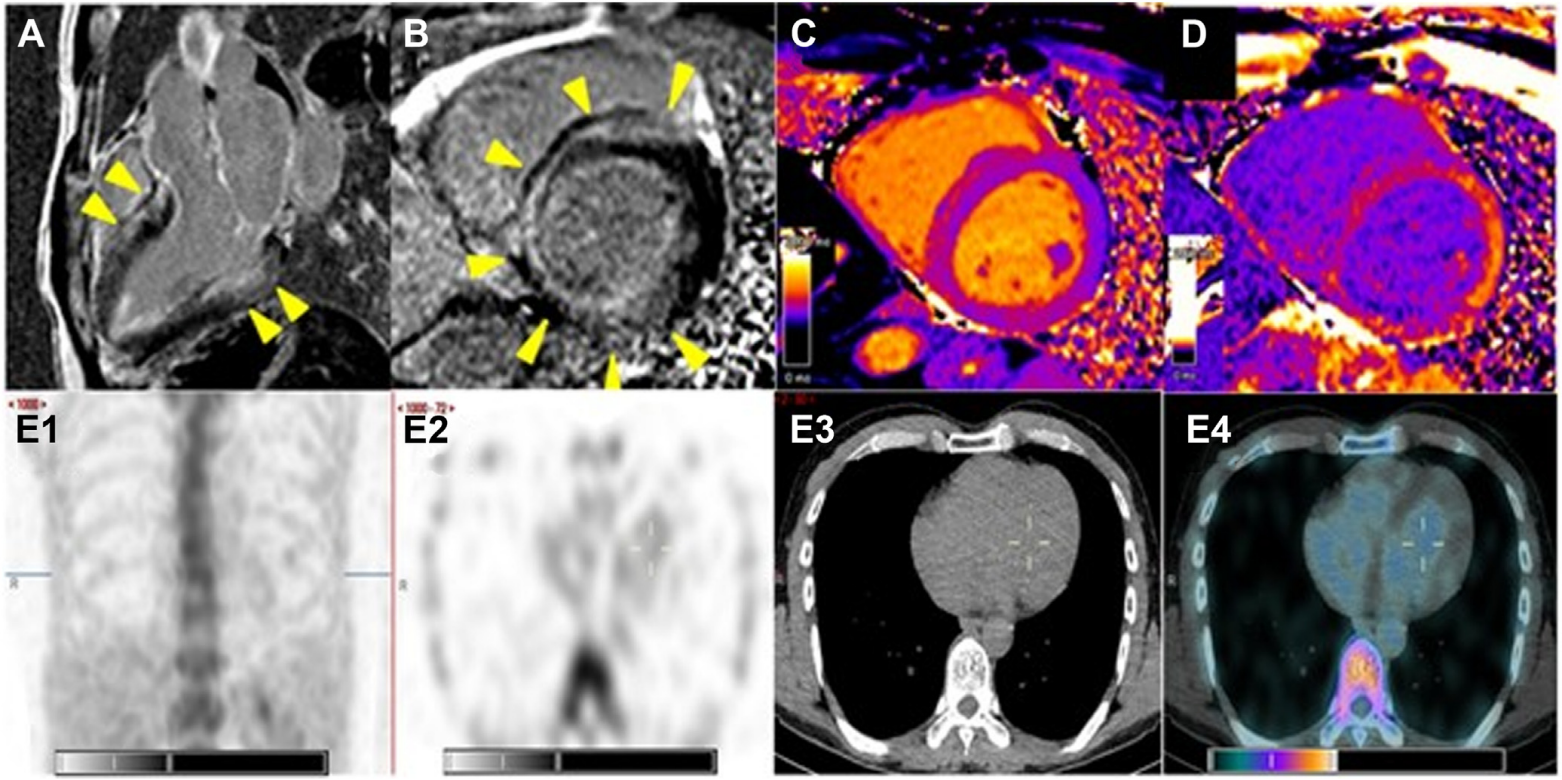
Postcontrast cardiac magnetic resonance with subendocardial to mid-wall late gadolinium enhancement of the basal myocardial segments in 3-chamber view (**A**) and basal short axis view (**B**). Precontrast T1-mapping (**C**) showed mildly increased T1 relaxation time in the basal segments, whereas the postcontrast T1 relaxation time was markedly reduced (**D**) allowing calculation of increased extracellular volume values between 37% and 40%. The ^99m^Tc-DPD scintigraphy showed no cardiac uptake, indicated as Perugini Grade 0 (E1-4). Trace uptake was noted at the bone level in E1. Imaging details: The cardiac imaging was performed using a Siemens Symbia Intevo 16 camera (Swedesboro, NJ) with a low-energy high-resolution (LEHR) collimator. The dose of Tc-99m-DPD was 8Mbq/kg with a scanning speed of 12 cm/min. Thorax static images (A/P) were acquired with 1000 kcts. SPECT/CT of the thorax used a 256 x 256 matrix, with 60 images per detector, each lasting 15 seconds in a noncircular orbit, continuous mode. Reconstructions were performed using FLASH 3D and XQUANT for quantification. The radiotracer injection was done at 10:13 AM, and image acquisition took place at 02:06 PM. SPECT/CT, single-photon emission computed tomography with a computed tomography scan.

He was admitted 6 months later to our emergency department for an unexplained syncope without prodroma. At admission, heart rate was 63 beats per minute, electrocardiogram result was in sinus rhythm, with a first-degree atrioventricular block (PR interval of 272 ms) and a QRS complex at 126 ms, indicative of a complete left bundle branch block. On echocardiogram, left ventricular wall thickness was mildly increased (maximal wall thickness 12 mm), and there was no increase in left ventricular mass index (101g/m^2^) or diastolic dysfunction. Left ventricular ejection fraction (LVEF) was normal, but longitudinal function was decreased, particularly in the basal segments, with a global longitudinal strain (GLS) of - 14.6%, raising the suspicion for cardiac amyloid infiltration (Fig. 1). On cardiac magnetic resonance (CMR), the LVEF was slightly reduced by 48%, without regional wall-motion abnormalities, but there was subendocardial to mid-wall late gadolinium enhancement in the basal to midventricular segments. By relaxometry, T1-relaxation time of the basal segments was mildly increased (1059-1081 ms), with a severe increase in extracellular volume (37%-40%, N < 28%; Fig. 2, A-D). After confirmation of the absence of clonal plasma cell process, we organized a technetium-99m (^99m^Tc)-diphosphonate (DPD) scintigraphy to make a formal nonbiopsy diagnosis of cardiac amyloidosis before starting an antiamyloid treatment. Surprisingly, it showed no tracer uptake (Perugini grade 0; Fig. 2E), and this result was in contradiction with those from echocardiography and CMR. Because of a high suspicion of early myocardial variant TTR infiltration, based on speckle-tracking echocardiography and CMR, the patient underwent myocardial biopsy, which confirmed the presence of amyloid deposits by Congo red staining (CRS). During the same procedure, the HV (His bundle to ventricular myocardium) conduction time was measured at 82 ms, confirming an infrahisian conduction disturbance, and a dual-chamber pacemaker was implanted.

## Discussion

Because of its high sensitivity for amyloid deposits in the myocardium,^99m^Tc-DPD/hydroxyl-methylene-diphosphonate (HMDP)/pyrophosphate (PYP) scintigraphy is a key imaging test to diagnose TTR cardiac amyloidosis. However, both false positive and false negative tests have been reported. Scintigraphy may be mildly positive in cases of amyloid light chain (AL) amyloidosis or other more rare types of amyloidosis (eg, ApoA, Aβ2M), or in case of hydrochloroquine cardiac toxicity. On the other hand, it may remain negative with certain rare TTR mutations such as pPhe84Leu (Phe64Leu), Ser97Tyr, and histologic confirmation may be needed in these cases.^2^

Histologic studies differentiated 2 types of myocardial deposits in hATTR. Type A deposits are composed of a mixture of protein fragments and intact proteins and are found in most ATTR cases (including wild-type ATTR); they are characterized by weaker CRS on histology and strong tracer retention on scintigraphy. On the other hand, Type B deposits, which are exclusively composed by unfragmented full-length proteins, are only found in relation with specific TTR mutations such as pVal50Met (Val30Met), pPhe84Leu (Phe64Leu), pGlu94Asp, or pTyr134Cys. They show brighter CRS and may be associated with normal scintigraphy.^3^ Indeed, Pilebro et al. showed that 44% of patients with pVal50Met (Val30Met) TTR had type B deposits, and none of those patients had positive ^99m^ Tc-DPD scintigraphy. Mean age at onset was significantly lower compared with patients with Type A deposits.^4^ This was confirmed in another study, reporting a significantly lower sensitivity of ^99m^Tc-DPD scintigraphy in early-onset (ie, < 50 years old) compared with patients with late-onset pVal50Met (Val30Met) hATTR (26.7% vs 73.3%, *P* = 0.005; all patients with increased wall thickness ≥ 13 mm).^5^ Furthermore, in their study, Musumeci et al.^6^ observed a very low sensitivity in detecting TTR cardiac amyloidosis concerning the pPhe84Leu (Phe64Leu) mutation. Only 2 of 19 patients (10.5%) exhibited high-grade myocardial bone tracer uptake (Perugini score 2). The authors explained potential factors contributing to this reduced sensitivity, which may include considerations such as the extent of amyloid deposits (early-stage disease), the timing of imaging after radiotracer injection, or the low affinity of amyloid deposits associated with the Phe64Leu mutation for the bone radiotracer.^6^

With the recent availability of effective anti-amyloid treatments, there is higher awareness of the importance of an early diagnosis of symptomatic hATTR amyloidosis. In this context, ^99m^Tc- DPD/HMDP/PYP scintigraphy has become a key imaging modality for timely detection of cardiac infiltration. However, despite the presence of clinically significant myocardial infiltration, some patients may not show tracer retention with scintigraphy. Such false negatives studies are not only related to a few rare mutations but also to the early-onset (minimal myocardial infiltration) form of pVal50Met (Val30Met) hATTR amyloidosis. Our case report again highlights the limitations of bone scintigraphy in detecting TTR cardiac amyloidosis linked to some mutations and emphasizes the importance of tailoring diagnostic approaches to ensure accurate diagnosis and, hence, suitable treatment.

In our patient, we initiated patisiran treatment on April 1, 2022. From a cardiologic perspective, the patient was last seen shortly after starting patisiran, but he missed 2 appointments thereafter. He did not exhibit any cardiorespiratory symptoms, and the medical status did not display signs of right or left heart congestion. The electrocardiogram was consistent with the one previously described. The echocardiogram revealed a normal ejection fraction as previously described, but we observed a normalization of the GLS, with an increase from –14% to –20%. Otherwise, we observed a fusion of the E and A waves making diastolic function difficult to assess, the left atrium was of normal volume, and there was no valvulopathy or pericardial effusion. As with the cardiologic follow-ups, he did not attend all the appointments, and the neurologic follow-up has therefore been challenging, but he was last seen on February 16, 2023. He reported an improvement anamnestically (resolution of pain, stable subjective scores); clinically (improvement in clinical scores, status, and vigorimetry); and even electrophysiologically (reduction in distal motor latency of the median nerve, good stability of other nerve conduction parameters). The infusions were generally well tolerated, except for some nausea and occasional diarrhea during the first 2 days after each treatment.

Finally, although the sensitivity issue related to scintigraphy in the case of a pVal50Met mutation has been reported previously, we believe that this case report still provides valuable insights to clinicians in the diagnostic and therapeutic management of cardiac amyloidosis for 3 reasons. First, even though the pVal50Met mutation is 1 of the most common mutations in the TTR gene, the risk of obtaining a false negative scintigraphy remains largely unknown among cardiologists, emphasizing the need to inform clinicians about this particularity. Second, we describe how an early form of pVal50Met hATTR can manifest, especially with conduction blocks without significant thickening of the myocardial wall or severe infiltration. Third, a recent opinion leader by Dorbala et al. recommends, for the purpose of early detection, starting with a scintigraphy for patients without heart failure but at risk of ATTR, especially in the context of familial amyloidosis.^7^ In our patient, this implies that the diagnosis would theoretically have been missed. Therefore, our case report stresses the need to reconsider the use of biopsy in the diagnostic management allowing for the rapid initiation of therapy.

## Conclusions

In patients with pVal50Met (Val30Met) TTR mutations and clinical suspicion of cardiac infiltration based on electrocardiography, echocardiography, or CMR, clinicians should not rely solely on ^99m^Tc-DPD/HMDP/PYP scintigraphy to rule out cardiac amyloidosis, and histologic confirmation should be considered.

**Novel Teaching Points**

• Understanding different types of myocardial deposits may improve interpretation of imaging studies and diagnostic tests. Our case report distinguishes between type A and type B myocardial deposits in hATTR amyloidosis. Understanding these differences is crucial because they can affect the results of imaging studies and the sensitivity of diagnostic tests.
• False negative results in scintigraphy present a diagnostic challenge in TTR cardiomyopathy. Our case report discusses the importance of recognizing false negative results, especially in specific TTR mutations, and highlights the limitations of certain imaging techniques.
• Multimodal diagnostic approaches may improve management strategies. Our case report emphasizes the importance of considering various diagnostic tools including echocardiography, scintigraphy, CMR and biopsy, to establish a personalized approach for accurate diagnosis and appropriate treatment.
